# Endoscopic submucosal dissection with metal clip closure for the treatment of a mid-esophageal diverticulum: a case report

**DOI:** 10.1055/a-2387-4290

**Published:** 2024-09-04

**Authors:** Zhenghua Wang, Bing Bai, Junmei Jiang, Hongwei Xu, Qi Zhao, Bin Li

**Affiliations:** 1 34708 Department of Gastroenterology, Shandong Provincial Hospital Affiliated to Shandong First Medical University, Jinan, China; 2 Digestive Diseases Hospital of Shandong First Medical University, Jining, China

Currently, the primary technique employed for endoscopic treatment of a mid-lower esophageal diverticulum is diverticular peroral endoscopic myotomy (D-POEM) 1 2 . The surgery dissects the diverticulum septum through a submucosal tunnel, thereby preventing food and liquid retention. However, the pouch persists and may continuously cause symptoms 3 . Since 2023, our team has been innovatively utilizing endoscopic submucosal dissection (ESD) with metal clips to treat Zenker's diverticulum and reporting corresponding complications 4 5 . Here we further applied the surgery to treat a mid-esophageal diverticulum and ultimately resolved the symptoms.

The case presented a 54-year-old man diagnosed four years previous with a 2.0-cm mid-esophageal diverticulum and adjacent mucosal high-grade intraepithelial neoplasia underwent endoscopic submucosal dissection (ESD) treatment and recovered satisfactorily. After discharge, the patient occasionally experienced 5- to 10-minute episodes of chest pain, tightness, and palpitations, relieved by rest. Angina was suspected, but percutaneous transluminal coronary angioplasty did not alleviate it. Symptoms worsened over the past month, becoming more frequent and severe. An upper gastrointestinal barium radiography revealed a 3.0-cm mid-esophageal diverticulum ( Fig. 1 ). Considering the symptoms are caused by the enlarged diverticulum pressing on the heart, D-POEM may result in esophageal cystic dilation, which remains near the heart and poses challenges in relieving symptoms. ESD was therefore performed ( Video 1 ).

**Fig. 1:**
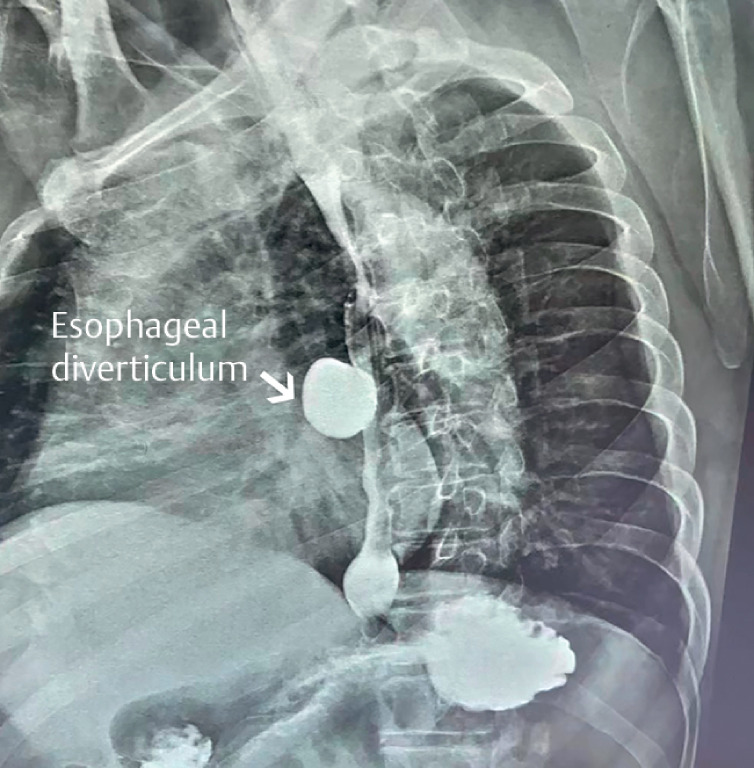
Preoperative upper gastrointestinal barium radiography showed a 3.0-cm mid-esophageal diverticulum.

Endoscopic submucosal dissection with metal clip closure to treat a mid-esophageal diverticulum.Video 1

After marking the surrounding mucosa of the esophageal diverticulum and submucosal injection, we gradually dissected the mucosal and submucosal layers inside the diverticulum ( Fig. 2 **a–d** ). Significant adhesions at the diverticulum base limited dissection space. The mucosal layer was excised in two stages using a snare trap ( Fig. 2 **e,h** ). Finally, we use through-the-scope twin clips (TTS-TCs) and multiple metal clips to close the diverticulum and repair the esophageal muscular layer ( Fig. 2 **f,g** ). The patient had no chest tightness or fever post-operation and experienced less severe chest pain than before. Repeat upper gastrointestinal radiography showed resolution of the esophageal diverticulum ( Fig. 3 ). The patientʼs preoperative symptoms disappeared and did not recur within a month on a regular diet.

**Fig. 2:**
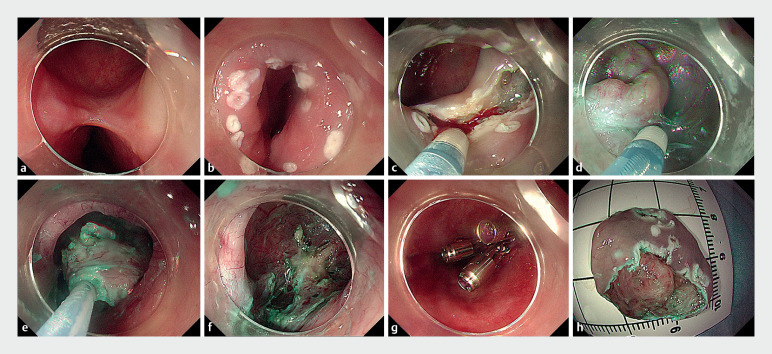
Endoscopic submucosal dissection with metal clip closure for mid-esophageal diverticulum. **a** The mid-esophageal diverticulum. **b** Marking the surrounding mucosa of the opening of diverticulum. **c** Circumferentially incising the mucosa around the opening of diverticulum. **d** Dissecting the submucosal layer inside the diverticulum. **e** Excising the mucosal layer with a snare trap. **f** Residual cavity of diverticulum. **g** Closure of the muscle layer at the opening of diverticulum. **h** Excised specimen of diverticulum.

**Fig. 3:**
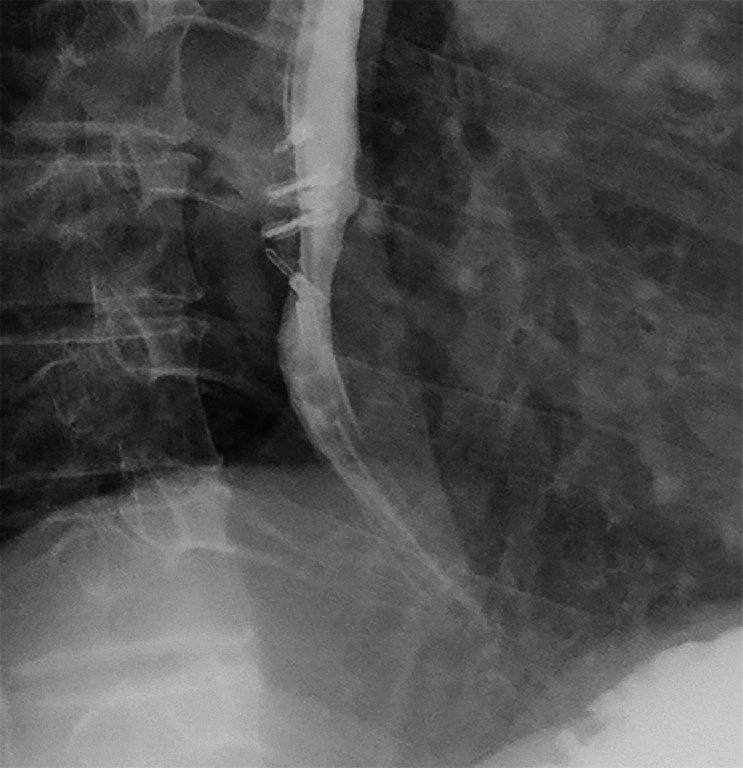
After 48 hours, upper gastrointestinal radiography showed resolution of the mid-esophageal diverticulum, and no contrast agent extravasation was observed.

Endoscopy_UCTN_Code_TTT_1AO_2AP

## Notes

Endoscopy E-Videos https://eref.thieme.de/e-videos
*E-Videos* is an open access online section of the journal *Endoscopy* , reporting on interesting cases and new techniques in gastroenterological endoscopy. All papers include a high-quality video and are published with a Creative Commons CC-BY license. Endoscopy E-Videos qualify for HINARI discounts and waivers and eligibility is automatically checked during the submission process. We grant 100% waivers to articles whose corresponding authors are based in Group A countries and 50% waivers to those who are based in Group B countries as classified by Research4Life (see: https://www.research4life.org/access/eligibility/ ). This section has its own submission website at https://mc.manuscriptcentral.com/e-videos .

## References

[1] Weusten BLAM Barret M Bredenoord AJ Endoscopic management of gastrointestinal motility disorders – part 2: European Society of Gastrointestinal Endoscopy (ESGE) Guideline Endoscopy 2020 52 600 614 32462649 10.1055/a-1171-3174

[2] Kamal F Khan MA Lee-Smith W Peroral endoscopic myotomy is a safe and feasible option in management of esophageal diverticula: Systematic review and meta-analysis Dig Dis Sci 2021 66 3242 3249 33123940 10.1007/s10620-020-06678-5

[3] Albéniz E Estremera-Arévalo F Gómez Alonso M Peroral endoscopic myotomy, septotomy, and restoration of esophageal lumen with over-the-scope clips: closing the circle of esophageal diverticula management Endoscopy 2022 54 E666 E667 35148540 10.1055/a-1724-6801

[4] Chen N Shi L Ge J Endoscopic mucosal dissection with metal clip closure for esophageal Zenker diverticulum: a case report (with video) Chin J Dig Endosc 2023 40 930 932

[5] Shi L Long F Xu H Chronic esophagotracheal fistula secondary to esophageal diverticulum successfully treated by endoscopic submucosal dissection and dual action tissue clip Endoscopy 2023 55 E1128 E1130 37875154 10.1055/a-2163-2050 PMC10597680

